# Supplementary material for: Transcriptome Analysis Reveals Vimentin-Induced Disruption of Cell–Cell Associations Augments Breast Cancer Cell Migration
Source: Cells. 2022 Dec 13;11(24):4035. doi: 10.3390/cells11244035 (PMC9776984; doi:10.3390/cells11244035)
Supplement: Supplementary file 1 [file cells-11-04035-s001.zip › DEGs and enrichment analysis.zip!/DEGs and enrichment analysis/WT_VIMvsVector KEGG/ALL/WT_VIMvsVector_ALL.pathway_map.zip!/WT_VIMvsVector_ALL.pathway_map/src/hsa00330.html]

hsa00330


- hsa:219

- Up regulated genes

ENSG00000137124(1.75993701688244)

- hsa:6303

- Down regulated genes

ENSG00000130066(-2.20463233119519)

- hsa:219

- Up regulated genes

ENSG00000137124(1.75993701688244)

- hsa:1152

- Up regulated genes

ENSG00000166165(2.27249671167479)

- hsa:65263

- Up regulated genes

ENSG00000104524(4.48097319445919)

- hsa:65263

- Up regulated genes

ENSG00000104524(4.48097319445919)

Close
